# New York Academy of Medicine—Section on Orthopedic Surgery

**Published:** 1892-06

**Authors:** 


### NEW YORK ACADEMY OF MEDICINE.—SECTION ON ORTHOPEDIC SURGERY.

*Stated Meeting, April 15, 1892.*

HENRY LING TAYLOR, M. D., Chairman.

Dr. L. A. SAYRE said that in his paper at the last meeting, he had referred to a case of hip disease which he had seen in consultation with Sir James Paget and Mr. Adams, of London, which it was generally considered could not recover without anchylosis and deformity. He was fortunate in having the opportunity of presenting this patient at this meeting. This man could place his feet on a table, could squat down, and, in fact, could perform every motion so well that it was difficult to see which had been the diseased hip.

### THE EFFECT OF PERSISTENT MOTION.

Dr. JOHN RIDLON exhibited a girl, nine years old, who had come to him at the Vanderbilt Clinic on April 23, 1891. Eight months previously she had received an injury to the right elbow, which was diagnosticated as a "fracture of the coronoid process of the ulna, and a dislocation backwards of the radius and ulna." She was attended by a well qualified practitioner of this city. The arm was immobilized for about four weeks, and then passive motion was commenced. Twice daily the forearm was flexed and extended on the arm to the limits of tolerance, and twice weekly, under an anesthetic, the forearm was flexed and extended to the normal limits of motion. This treatment was faithfully continued for seven months, during which time the range of motion gradually became more restricted, the joint more and more swollen, and more painful under the attempts at motion. Examination showed the forearm flexed on the arm to a right angle, much swelling about the joint, enlargement of the superficial veins, and atrophy of the muscles of the arm and forearm. The swelling had a pulpy feeling, but no point of fluctuation could be detected. The bony points were so obscured that the exact nature of the injury could not be determined. There was no motion of the joint, and attempts at motion caused pain and developed intense muscular spasm.

The treatment adopted was as follows: The head was bent down, and the wrist put in a "halter" made out of a roller bandage, knotted around the wrist and neck. The slack of this was taken up as the rigidity yielded, and at the end of two weeks the joint could be completely flexed. In this position, the joint was held without motion being once permitted or tested for eleven months. The pain disappeared, the swelling gradually subsided, and, when the halter was removed, there was found to be free, painless motion, from a right-angle to normal flexion. Since then there has been no treatment, and the range of motion in the direction of extension is gradually increasing.

Dr. W. R. Townsend said that this girl had been brought to the Hospital for Ruptured and Crippled, about two years ago, by her attending physician, who said that passive motion had been made, under ether, about three times a week since the fracture, to prevent anchylosis. Dr. W. T. Bull, who saw the case in consultation, agreed with the speaker in advising rest. The attending physician dissented from this view, but finally said he was willing to give the joint rest for a limited time, if he were relieved from all responsibility as to the result. The case was accordingly treated in the hospital with a plaster-of-Paris splint for about four weeks, when the mother objected to a continuance of this treatment. Dr. Bull again saw the case in consultation, and the opinion was then expressed that there was a beginning osteitis, and that if motion were kept up, the child would undoubtedly have a stiff elbow. The patient and doctor dissented again, and wished passive motion made, so she was then discharged from the hospital out-patient department.

Dr. S. KETCH said that if the arm were moved beyond a certain point, especially in rotation, there is reflex spasm, and he thought there was still some active disease in the elbow joint. He asked if the halter allowed of pronation and supination.

Dr. RIDLON replied that the halter did not prevent these motions, but, so far as his experience with it had gone, when properly applied under the clothes, the children, as a matter of fact, do not attempt to make these motions.

Dr. RIDLON also presented a case for diagnosis :

A man, thirty-four years old, came to him at the Vanderbilt Clinic, on February 15, 1892. For two weeks he had been stooping and stiff in the lumbar spine, with pain in the back and lower abdomen, and, at times, down the front of the thighs. Seven years ago, he had a similar attack, at which time, after suffering for four weeks, he went to the dispensary of an orthopedic institution in this city, where the diagnosis was made of Pott's disease, and a Taylor spinal brace applied. He remained in bed for two months, wearing the brace, but without any relief. He was then admitted to the St. Francis Hospital, where a blister was applied, and the pain immediately relieved. At the end of two weeks, he was quite well again, and remained so up to the present attack. Examination revealed the whole lumbar spine curved backward and rigid ; there was psoas contraction on the left side, but none on the right ; and there was a doubtful fulness in the left iliac fossa. He was treated with anti-rheumatic remedies, and soon showed improvement, and in the course of a few weeks felt entirely well. There is now no spinal curvature, no rigidity, no psoas contraction, and the patient is quite well, except that at times, after long sitting over a bench at his work, he feels the back somewhat stiff.

### AN INEXPENSIVE HEAD SUPPORT.

Dr. ROYAL WHITMAN exhibited a support which he had devised for a child with mid-dorsal disease, in whom there was a tendency for the shoulders and the whole body to droop forward. The support consisted of a curved piece of steel attached to the back of the brace used in connection with lateral pads for holding the shoulders back, a form of apparatus which he had already exhibited and described.

Dr. V. P. GIBNEY then exhibited a series of operative cases.

### ANCHYLOSIS OF HIP AFTER TYPHOID FEVER.

The first case was that of a boy, eleven years of age, whose trouble dated back to an attack of typhoid fever, in November, 1889. He was admitted to the hospital July 14, 1890, and at that time his general condition was fair; there was a long and deep cicatrix over the right hip. There was about half the normal motion. The left hip was held flexed at 135°, and there was much spasm. His mother died of phthisis, but the other members of the family were healthy. One month later, the right limb could be extended to 180°, and the left limb to 170°, with little or no motion at the knee. He was then allowed out of bed, wearing a brace on the left side, and a high shoe on the right. On November 19th, there was some tenderness around the joint, but none in the joint. The brace was left off, and he was given cod liver oil, and allowed to walk around. On January 16, 1891, the right thigh was flexed on the abdomen. The adhesions were broken up by manual force, and in doing so, a "snap" occurred. The tensor vaginæ femoris and the fascia were divided subcutaneously, when the deformity yielded visibly. The limb was then hyper-extended, abducted, and rotated outward, and put in plaster-of-Paris. On February 11th, as the thigh was flexing, the limb was placed on an inclined plane, with a weight and pulley, and this treatment was continued for nearly one month. The left hip was then extended at an angle of 158°, with practically no motion, and the right hip at 170°. On June 13th, the right thigh could be completely extended; the left to 140°. On October 23d, under ether, it was found that the hip could not be fully extended, so the tense bands attached to the anterior superior spine were divided, and then the limb almost completely extended, and put in plaster-of-Paris. On January 18, 1892, he was etherized, and a femoral osteotomy performed, and the limb hyper-extended and put in plaster, and by February 17th there was firm union, the limb was extended at an angle of 175°, and with 5° of motion. The ordinary hip splint was then applied. On March 5th, the limbs were parallel, union was very firm, and his general health was good. On April 6th, he was allowed to walk about without any support.

### EXCISION OF ONE HIP IN A CASE OF DOUBLE HIP DISEASE WHERE SACRO-ILIAC DISEASE WAS FIRST DIAGNOSTICATED.

The next case, a boy, nine years old at the time of his admission, on January 22, 1890, had a good family history, and his hip disease was attributed to a fall in September, 1889. Before coming to the hospital, he had been treated in a dispensary for sacro-iliac disease. At the time of admission, the thigh could be flexed to 60°, and extended to 180°; all the movements were restricted by pain. On March 4, 1890, both thighs and knees were flexed, and there was much reflex spasm about the right hip, and somewhat also about the left. The hips and knees were extended, and the limbs made parallel, and they were then put up in plaster-of-Paris spica. About this time, the temperature began to rise to 102° every night, and to fall in the morning to 98.8°, and there was marked tenderness in the right hip. On March 29th, there was fulness and deep fluctuation in the upper fourth of the thigh, and a needle withdrew some pus and blood. A hip splint was applied, and the child put to bed. On April 8th, a double spica with traction was applied. On September 3d, about seven ounces of pus were removed from the right thigh by aspiration, and on the 30th of the same month the child was etherized, an old sinus curetted and enlarged, and a counter opening made. The head and neck of the femur and the acetabulum were found to be diseased. The temperature remained high until October 15, 1890, when a large abscess was found over the trochanter major, which did not communicate with the sinus. On January 28, 1891, after poulticing, the abscess opened spontaneously. On February 10th, the child was etherized, and excision performed. The head and neck were curetted, a counter opening made, and a double spica applied. The dressings were changed every third or fourth day, peroxide of hydrogen solution being used to wash out the cavity. By July 2d, the excision wound was almost completely closed; there was some infiltration about the joint, but very little spasm and tenderness, and the limb could be extended to 180°, and flexed to 160°. By September 16th, flexion had been increased about 10° in the right limb, and the left limb could be extended to 180° and flexed to 135°, motion being slightly restricted by spasm. On December 16th, a jointed splint was applied, upon which he walked around with comfort. On February 27, 1892, it is noted that there is a fair amount of motion at the right hip, without pain, the other limb remaining unchanged since last note.

### EXCISION OF HIP WITH FAIR RESULT, SCARCELY FINAL.

The next case was a girl who was eleven years of age at the time of her admission on June 6, 1889, with hip disease of six months' duration. Her mother had died the previous year of some pulmonary affection. The child was poorly nourished, and could hardly stand ; the right limb could be flexed to 135°, and abducted one-fourth of an arc, and the left correspondingly adducted ; there was marked tenderness and spasm. On July 31, 1889, a small quantity of pus was removed by aspiration, and the hip splint was applied. On December 9th, of the same year, the limb was much abducted, and there was some infiltration in the upper part of the thigh. On May 13, 1890, the hip was very acutely inflamed, and the child had night cries, and tonics and stimulants were ordered. On December 2d, there was very little discharge, and the wounds looked healthy. On February 12th, four sinuses were discharging freely, and there was considerable burrowing of the pus. On April 24, 1891, excision was performed, involving three of the sinuses. The capsule was practically gone, and the head of the bone carious, and there was a shell of exfoliated bone. After dividing the bone at the neck, it was found that the disease extended into the shaft, so one and a half inches were removed, and the parts thoroughly curetted, packed, and drained. On July 18th, the child was etherized, and some sinuses again scraped out. On July 27th, there was ten degrees of motion, and the limb was extended to 170°. By August 20th, there was scarcely any discharge, no tenderness, the limb could be extended to 180°, and there was 20° of motion. On February 18, 1892, it was noted that the angle of greatest flexion was 100°, and greatest extension, 180° ; there was no spasm or pain on manipulation. At present she is wearing a caliper splint, the right thigh can be flexed nearly to ninety degrees, abduction is quite good, and extension is nearly perfect.

### CASE FOR DIAGNOSIS, PROBABLY SUBACUTE RHEUMATISM.

A little boy was next exhibited, who had been sent to the out-patient department of the hospital about three weeks before for a diagnosis. For three months he had complained of pain and stiffness in the hips and knees. He had the walk of hip disease, with slight spasm, and slight limitation of motion, and it was a question whether there was really incipient hip disease, or whether there was a neurotic element in the case. He was given the salicylates internally. Soon after he began to complain of pain in the knees and ankle, while the hips had improved slightly. He had been in the hospital only two days, but in this time there had been some improvement. There was evidently pain, and, undoubtedly, also an inflammatory process, as on several occasions his temperature reached 101 or 103°.

ATONIC KNOCK-KNEE ; FAILURE TO RELIEVE BY APPARATUS ; SUPRA-CONDYLOID OSTEOTOMY ; SUBSEQUENT SYNOVITIS ; LATER STILL, APPARATUS ; RESULT, VERY UNSATISFACTORY.

The fifth case was a girl who first came to the dispensary on January 1, 1888, when fourteen years old. She then had right knock-knee, which was treated with a Thomas knock-knee splint. The brace was worn for about one year, and then was removed, and the shoe built up on the inner side. On August 5th, the knee had again become relaxed, and plaster-of-Paris was applied. On October 21st, there was a synovitis of the right knee, and the girl was admitted to the hospital shortly afterward. At this time both knee joints were relaxed, and could be hyper-extended ; there was flexion to about 120°. The knee joint was distended with fluid, and there was some joint tenderness. On November 14, 1889, about twenty ounces of clear fluid were drawn from the joint, which was then irrigated with carbolic acid solution, 1.40, and the limb put up in plaster-of-Paris. On January 30, 1890, there was again some effusion into the joint, and she walked with some stiffness. There was flexion to 140°. On February 27th, she was discharged unimproved, and on March 31st, she was re-admitted. At this time there was marked lateral improvement, the tibia could be abducted so as to make an angle of 153° with the femur, and adducted to 175° ; the knee could be flexed to 110°. There was also much pain in the muscles about the right hip and thigh, and considerable tenderness in the iliac fossa, with prominence just in front of the great trochanter. On September 18, 1890, the pain had disappeared from the hip, and she was wearing a Knight posterior knee brace. She was discharged from the hospital then, but again admitted on May 27, 1891. Finally a supra-condyloid osteotomy was performed from the outer side, and the knee over-corrected, and put up in plaster for awhile. At present she is wearing a splint without motion at the knee, and, although she walks well, there seems to be no prospect of improvement.

SOME OF THE INDICATIONS FOR OPERATIVE INTERFERENCE IN ORTHOPEDIC SURGERY.

A paper with this title was read by Dr. V. P. GIBNEY.

The paper dealt first with the range of orthopedic surgery as held by the majority of surgeons practising this specialty throughout the world. It commented on the brilliant results obtained by general surgeons in many cases that were strictly orthopedic. It emphasized the importance of supplementing operative procedures with mechanical appliances.

It was suggested that orthopedic surgery might be advanced if one devoted as much time to the clinical history and the pathology of the disease which produces deformity, as one does to the devising of splints and modifications of splints. The importance of devising splints to suit individual cases, and to meet certain conditions, was regarded as an important part of orthopedic surgery.

It was stated that the orthopedic surgeon seldom has an opportunity of putting a splint on a case in the very first stage of the disease; that many of the cases of what are called early hip disease were not early cases, but that deformity had already arisen when they came to mechanical treatment. The same was true of Pott's disease of the spine.

Some of the indications for operative interference were mentioned as correction of deformity in these early hip cases by manual force under ether, by division of tendons and muscles, if the correction were difficult; and, in the advanced cases, where the disease was fully arrested, osteotomy below the trochanter minor was suggested as a valuable addition to our therapeutics.

With regard to abscesses, it was urged as good orthopedic surgery to make incision, if four or five aspirations failed to relieve. It was further suggested that old sinuses and pockets of pus should be treated by operative interference.

Operative interference in spinal disease was not recommended, except where a severe trauma had fractured the lamina, and where pressure had resulted. In these cases, laminectomy was advised, but it was suggested that, in many instances of this kind, the ordinary mechanical treatment proved of valuable service.

In disease of the knee, partial arthrectomy was advised, in preference to complete arthrectomy or to excision, especially in children. In the internal derangements of the knee, operative interference was advised, rather than the prolonged use of apparatus and fixation splints. In synovial disease, pure and simple, an occasional aspiration of the joint, with strapping, was regarded as good orthopedic surgery.

DISCUSSION.

Dr. L. A. Sayre said that the paper covered too broad a field to admit of discussing it in detail, but in general the author had expressed his own views most accurately.

Dr. JOHN RIDLON did not consider that the element of time was very important, except in those exceptional cases where it was a difference between a few weeks and several years. On the principle of leverage, he had been able to reduce the deformity in some of the very worst cases of hip-joint disease in a few hours, or a few days, as safely by mechanical means as by operation. It was only the question between a few days following an operation, and a few days more with mechanical treatment, and, under these circumstances, we should not think of doing a cutting operation. In all cases of disease of the hip or of the knee, leverage reduction will accomplish the result as well as operation.

Dr. S. KETCH said that while theoretically the orthopedic surgeon should be a good general operating surgeon, in practice he is not frequently called upon to perform operations, and hence, could not be expected to be as skilful manually as surgeons who are constantly operating, and on this account, a natural division of labor was founded. He inferred from the paper that the author must have met with a class of cases in which it was unusually difficult to reduce the flexion, for, as a rule, there was no special difficulty about reducing this deformity, provided sufficient time were allowed. Forcible leverage or stretching adds an unnecessary risk, as there is no way of accurately gauging the amount of force employed, and hence, there is danger of inflicting traumatism which will result in lengthening the course of the disease, and causing a speedy return of the deformity.

Dr. W. R. TOWNSEND thought there was one class of cases in which mistakes are likely to follow mechanical treatment, but which yield brilliant results after operation, viz., the so-called periarticular abscesses. Such an abscess situated outside of the hip-joint often gives rise to symptoms simulating hip disease, and if not treated by operation, there is great danger of the abscess opening into the joint.

Dr. L. W. HUBBARD endorsed what Dr. Ketch had said about the treatment of deformity in the early stages, and he had found in his own experience, that the reduction was usually quite rapid. He had never seen a case of hip disease in any stage, where there was motion, in which the deformity could not be reduced by position and traction in a short time, usually not over six to eight weeks. He could not see the force of the remarks just made about periarticular abscesses, for they are just as likely to open externally as internally, and, as a rule, they heal quickly without operation.

Dr. H. W. BERG said that, had it not been for careful attention to mechanical details, such important orthopedic appliances as the plaster jacket, the long splint, and the Taylor brace, would not have been known, yet orthopedic surgery should be broad enough to include within its scope both mechanical and operative treatment.

Dr. N. M. SHAFFER thought that many of the conditions described should necessarily come under the care of the general, and not the orthopedic, surgeon. We are all agreed, however, that the orthopedic surgeon should be competent to perform all the operations of general surgery, just as he should be able to diagnosticate typhoid fever, or the exanthemata, etc. But it did not follow because the orthopedist is prepared to perform these operations, or to diagnosticate the diseases coming under the care of the physician, that he should do either the one or the other, unless circumstances made it absolutely necessary for him to do so. Dr. Shaffer would have orthopedic surgeons devote themselves to the science and art of the mechanical treatment of deformities, using operative surgery as an adjunct to the mechanical work, rather than, as many of us are prone to do, make the mechanical part a sort of kite-tail to operative surgery. There is so much to be learned, and so much to be developed, in the continually broadening field of mechanical treatment, that there seems to be no excuse for the present tendency of orthopedic surgery to invade the well-recognized boundaries of general surgery. The tendency ought to be the other way, if orthopedic surgery is to succeed as a specialty.

Dr. R. H. SAYRE said that orthopedic surgeons should be competent to take charge of a case from beginning to end, whether it required mechanical or operative treatment. Limiting orthopedic surgery to the use of apparatus is like limiting the oculist to the application of glasses for the correction of refractive errors.

Dr. ROYAL WHITMAN said that he was unable to see the force or the application of Dr. Shaffer's remarks on the paper of the evening. A specialist was so by reason of the class of cases he treated, not because of the means he employed. The broadening field for this specialty was the study of the etiology, development and cure of deformities; the study of the course, complications, and ultimate results of joint diseases. Treatment must vary with the social environment of the patient, the severity and duration of the disease or deformity, and the most successful surgeon was the one who could best adapt the means to the end to be accomplished.

Early diagnosis and efficient treatment would, to a great extent, obviate the necessity for operation, and it was proper for one who could select his cases, to devote himself exclusively to mechanical treatment. On the other hand, many chronic and desperate cases of disease and deformity were brought to the institution with which he was connected. These patients would be neglected at home, and rejected at general hospitals. Mechanical treatment alone in this class was ineffective, unless supplemented by operation, which was often a necessary and a life-saving procedure. This exaltation of mechanics was opposed to the best interests of the patients, since, in the minds of many, mechanical and operative treatment, which are mutually dependent, were contrasted and opposed to one another; thus patients, on the one hand, were subjected to early and unnecessary operation, and afterwards neglected, and, on the other, the benefits of legitimate surgical interference were not appreciated. Why a broader and, as it seemed to him, more rational view of the subject need prevent the study and appreciation of mechanical supports, was not apparent. Believing that disease was to be treated in its entirety, and not in phases, he was unable to accept the limitations that Dr. Shaffer would impose on the future development of orthopedic surgery.

The CHAIRMAN said that if the orthopedic surgeon must be familiar with operative methods, as undoubtedly he must, he should also be a competent neurologist, for just as serious mistakes would follow ignorance of this subject as ignorance of operative surgery. Certain limitations are naturally placed upon one's practice, depending upon whether it is private, or dispensary, or hospital practice; for, in the latter, it is often not the best ultimate result, but the best that can be obtained within a limited time, or with limited means, that must decide the plan of treatment to be adopted. The author's directions in regard to the reduction of the deformity in joint disease, and especially in certain stages of hip disease, while perhaps successful with him, would be exceedingly dangerous if followed by the general practitioner.

Dr. GIBNEY, in closing the discussion, said that the great drawback to letting the general surgeon operate upon our cases, is that we frequently lose sight of them, and they are accordingly allowed to go without the use of protective apparatus, and that careful treatment after operation, which is necessary in all these cases to insure good results.

---

NITRIC ACID IN MALIGNANT PUSTULE.—Dr. von Ruck reports (*N. C. Med. Jour.*) a case of malignant pustule upon the forefinger of the left hand, which came under his notice before general infection had occurred. The pustule presented the characteristics as described in medical literature; the source of infection was, however, obscure. The patient had been handling cattle, and the day before its occurrence had cleaned out with his hands the mangers of his cattle barn. Microscopical examination of the discharging serum from the vesicle was negative, but upon consultation it was determined to destroy the vesicle and its base with fuming nitric acid, and to make culture experiments with the discharging serum. The culture, in a moist brood oven, with a temperature of 100° F., succeeded, and the diagnosis was confirmed by the characteristic growth of the anthrax bacillus. The patient made a prompt recovery.—*St. Louis Med. and Sur. Jour.*

